# Factors affecting delays in seeking treatment among malaria patients during the pre-certification phase in China

**DOI:** 10.1186/s12936-024-04892-4

**Published:** 2024-03-11

**Authors:** Lianyu Jia, Xiaoyu Chen, Zhanchun Feng, Shangfeng Tang, Da Feng

**Affiliations:** 1 School of Medicine and Health Management, Tongji Medical College, Huazhong University of Science and Technology, Wuhan, 430030 Hubei China; 2 School of Pharmacy, Tongji Medical College, Huazhong University of Science and Technology, Wuhan, 430030 Hubei China

**Keywords:** Delay in seeking treatment, Imported malaria, Patient delay, Doctor delay

## Abstract

**Background:**

Delays in malaria treatment can not only lead to severe and even life-threatening complications, but also foster transmission, putting more people at risk of infection. This study aimed to investigate the factors influencing treatment delays among malaria patients and their health-seeking behaviour.

**Methods:**

The medical records of 494 patients diagnosed with malaria from 6 different malaria-endemic provinces in China were analysed. A bivariate and multivariable regression model was used to investigate the association between delays in seeking treatment and various factors. A Sankey diagram was used to visualize the trajectories of malaria patients seeking medical care. Total treatment delays were categorized as patient delays and doctor delays.

**Results:**

The incidence of total delays in seeking malaria treatment was 81.6%, of which 28.4% were delayed by patients alone and 34.8% by doctors alone. The median time from the onset of symptoms to the initial healthcare consultation was 1 day. The median time from the initial healthcare consultation to the conclusive diagnosis was 2 day. After being subjected to multiple logistic regression analysis, living in central China was less likely to experience patient delays (OR = 0.43, 95% CI 0.24–0.78). The factors significantly associated with the lower likelihood of doctor delays included: age between 30 to 49 (OR = 0.43, 95% CI 0.23–0.81), being single/divorce/separated (OR = 0.48, 95% CI 0.24–0.95), first visiting a county-level health institution (OR = 0.25, 95% CI 0.14–0.45), first visiting a prefectural health institution (OR = 0.06, 95% CI 0.03–0.12) and first visiting a provincial health institution (OR = 0.05, 95%CI 0.02–0.12). Conversely, individuals with mixed infections (OR = 2.04, 95% CI 1.02–4.08) and those experiencing periodic symptoms (OR = 1.71, 95% CI 1.00–2.92) might face increased doctor delays. Furthermore, higher financial burden and complications were found to be associated with patient delays. Doctor delays, in addition to incurring these two consequences, were associated with longer hospital stays.

**Conclusion:**

There was a substantial delay in access to health care for malaria patients before China was certified malaria free. Region, marital status, periodic symptoms and the level of health institutions were factors contributing to delays in treatment-seeking among malaria patients.

**Supplementary Information:**

The online version contains supplementary material available at 10.1186/s12936-024-04892-4.

## Background

Despite substantial global efforts to combat malaria, with some notable successes, malaria remains a major public health concern worldwide [1, 2]. The number of malaria cases was still on the rise globally in 2022, with about 249 million cases, an increase of 5 million cases compared with 2021 [3]. Additionally, imported malaria cases can occur anywhere in any country, potentially exacerbating the global malaria burden further [4–6].

Malaria used to be one of the oldest-known, most widespread and severe infectious diseases in China, posing a significant threat to public health and life safety for a long time [7]. In the 1950s, the annual incidence of malaria in China was as high as 1229/100,000, with the case-fatality rate of approximately 1% [8]. However, in 2010, China launched the National Malaria Elimination Action Programme (NMEAP) and embarked on the phase of malaria elimination. As a result, the number of cases dropped to around 5000 per year [9]. China achieved the milestone of zero indigenous malaria cases for the first time in 2017, and received official certification as malaria-free from the World Health Organization in 2021 [10].

Nevertheless, achieving the goal of malaria elimination does not mean that there is no threat of malaria. Any slackening in prevention and control efforts could lead to a rapid resurgence of malaria. In areas where malaria transmission has disappeared, individuals are becoming susceptible to the parasite and will not have any naturally acquired immunity to protect them from the risk of this potentially life-threatening disease. Furthermore, with the growing frequency of population movements, travelers returning from malaria-endemic regions may reintroduce cases [11, 12]. The experiences of countries like Greece and Italy, which faced the resurgence of local malaria following imported cases after achieving domestic elimination, serve as a stark reminder. Currently, there are more than 2000 imported malaria cases in China each year, posing a long-term challenge in maintaining the achievements of malaria elimination [13, 14].

The World Health Organization emphasizes the importance of early diagnosis and prompt treatment within 24 h after the onset of symptoms. Delays in seeking treatment for malaria can lead to disease progression, an increased risk of complications, and even ongoing transmission of the malaria parasite [15, 16]. Although China has been certified as malaria-free, the main challenge in managing malaria cases remains its delayed recognition and prompt interventions [17]. The health-seeking behaviour of malaria cases can be conceptualized in two steps: perceiving a need for treatment and engaging in treatment [18]. Delays at either of these steps can originate from patients themselves or healthcare providers. Few literature has focused on the delays in malaria treatment in regions like the Thai-Myanmar border and northern Ethiopia [19, 20], identifying determinants such as testing facilities, clinical expertise of doctors, and patient education levels. However, there is a lack of research on the determinants of delayed treatment-seeking behaviour and the types of delays during the pre-certification phase in China. Similar to China, some countries face the risk of imported malaria after eliminating indigenous malaria, and understanding the factors affecting treatment delays in China could provide valuable insights for other countries. Additionally, studies on the trajectories of seeking malaria treatment are limited, and an analysis of patient flow may facilitate the effective and timely implementation of control interventions in malaria management. This study selected 494 confirmed malaria cases from 6 malaria-endemic provinces in China, aiming to characterize profiles of delays and identify predictors of both patient delays and doctor delays. These findings might contribute to enhancing early healthcare-seeking behaviour for the prevention of re-establishment of malaria.

## Methods

### Study design and site

Based on the data provided by the National Health Commission, a two-stage cluster sampling method was used to investigate malaria cases and patient visits reported by health institutions from January 1, 2014 to December 31, 2016. The sampling design was based on previous work [21]. A total of 1633 malaria cases were collected from 63 hospitals in the sampled provinces according to the workload of malaria treatment. Due to limitations related to the availability and completeness of cases, 494 malaria patients admitted to 26 hospitals were finally included in this analysis after careful review of the information on each malaria case extracted.

A cross-sectional study was conducted in 6 representative provinces in China with a high prevalence of malaria: Zhejiang and Jiangsu in eastern China, Henan and Anhui in central China, as well as Yunnan and Sichuan in western China (Additional file 1). Each of the two selected provinces had the highest incidence of malaria in their respective regions. Zhejiang and Jiangsu provinces are located in the southeast coast of China, with well-developed economies and close exchanges of inbound and outbound personnel. Henan and Anhui, as inland provinces, have large rural populations and frequent population movements. Sichuan and Yunnan are adjacent to each other in the north and south, and Yunnan shares borders with malaria-endemic countries such as Laos, Myanmar and Vietnam.

### Data collection

The study population consisted of 494 patients diagnosed with malaria at the hospitals during 2014–2016. All cases in the sample identified as imported malaria. The presence of *Plasmodium* was diagnosed by microscopy, rapid diagnostic tests (RDT), nested polymerase chain reaction (nPCR), or a combination of all. Data were extracted from the medical records of malaria patients provided by health institutions, and contained a large number of demographic characteristics such as gender, age, marital status, occupation, and residence. Moreover, clinical and epidemiological data were obtained for each case, including the species of *Plasmodium*, complications, date of symptoms onset, and date of diagnosis and treatment. Medical costs, length of hospital stay, and complications were considered as outcome variables. The median cost of the sample was converted to United States Dollar (USD) according to the average exchange rate in 2014 (1 USD = 6.14 RMB), and USD 1018 was calculated as the critical value to judge the level of economic burden of malaria patients. Similarly, the median hospitalization time of the sample was 6 days, which was used as the critical value to judge the length of hospital stay of malaria patients. All data were anonymized.

### Definitions

Patient delays were defined as the time interval of more than 1 day between the onset of symptoms and the initial healthcare consultation. Doctor delays were defined as the time interval of more than 1 day between the initial healthcare consultation and the conclusive diagnosis. Total treatment delays were defined as the sum of patient delays and doctor delays. The unit of measurement for delay was in days.

In China, health institutions encompass provincial, prefectural, and county hospitals, township health centers, and village clinics, primarily tasked with providing treatment services [22]. It is mandated by health administration that all levels of health institutions possess fundamental capabilities for malaria diagnosis and treatment. Additionally, health institutions include the Centers for Disease Control and Prevention (CDCs), which are categorized into national, provincial, prefectural, and county levels. The CDCs have the capabilities to detect malaria and distribute anti-malarial drugs in accordance with China’s 2010 NMEAP strategy. Generally, health institutions with higher administrative levels tend to provide better medical services [23].

### Statistical analysis

All statistical tests were performed using the STATA 17.0 software package. Descriptive statistics were used to summarize the sociodemographic characteristics. When measurement data were skewed, they were expressed as “median (quartile) [M (Q1, Q3)]”, and count data were expressed as “rate or component ratio (%)”. Chi-square (χ2) test or Fisher’s exact test was utilized to compare differences between categorical variables. Normality tests were performed for continuous variables, and bivariate analysis was employed to identify important factors affecting patient delays, doctor delays, and total delays in malaria treatment. Multiple logistic regression was then calculated to find out correlations between relevant characteristic factors and delays in seeking treatment among the subjects. A two-sided *P* value less than 0.05 was considered statistically significant. The Sankey diagram was used to map the treatment-seeking pathways of malaria patients. This specific type of flow diagram uses varying path widths to represent quantities.

### Ethical approval and consent

The study was approved by the Ethics Committee of the Tongji Medical College of Huazhong University of Science and Technology (IORG0003571). Written permission to access and analyse the research data was granted by the National Health Commission of the People’s Republic of China and the administrators of each hospital. Patient information was anonymized and de-identified.

## Results

### Sociodemographic characteristics

A total of 494 participants were investigated (Table 1). The majority of malaria patients (86.9%) were below 50 years old, with a significant concentration (64.0%) in the 30–49 age group. The gender distribution showed that 485 (98.2%) were male, while 9 (1.8%) were female. Nearly eighty percent (79.8%) of the patients were currently married. In terms of occupation, 170 (34.4%) were farmers, 96 (19.4%) were workers, 89 (18.0%) were professionals, and the remaining (28.2%) had other types of occupations, like self-employed entrepreneurs, daily laborers or retirees. Over half of the patients (62.2%) originated from rural areas. Patients from eastern, central, and western China accounted for 13.6%, 62.6%, and 23.9%, respectively. Regarding medical insurance coverage, 14.2% of malaria patients were covered by employee medical insurance, mainly in the public sector and state-owned enterprises. Two hundred and fifty-three (51.2%) unemployed individuals (including children, the elderly and other unemployed residents) were covered by resident medical insurance. In addition, 30% of patients had no health insurance and the insurance status of the remaining (4.6%) was unknown.

More than half of patients (62.4%) were infected with *Plasmodium falciparum*, while 19.8% had *Plasmodium vivax*/*Plasmodium malariae*, 3.0% had *Plasmodium ovale,* and 14.8% had mixed infections. Almost all patients (99.4%) presented with fever, and other symptoms such as chills, sweating, periodicity, anaemia, and splenomegaly accounted for 42.3%, 38.7%, 24.9%, 8.3%, and 1.4%, respectively. After the onset of symptoms, the initial health institutions chosen by malaria patients was county-level health institutions (32.0%), followed by village clinics/township health centers (30.0%), and prefectural health institutions (29.3%). Only 43 (8.7%) patients initially sought professional care in provincial health institutions.

### Factors affecting delays in seeking treatment

Significant factors influencing total delays in seeking treatment identified by the bivariate analysis were: being single/divorce/separated, being a farmer, living in Western China, infection with *Plasmodium vivax*/*Plasmodium malariae*, mixed infections, first visiting a county-level health institution, first visiting a prefectural health institution, first visiting a provincial health institution, fever, and having periodic symptoms (Table 1).

**Table 1:** Factors associated with total delays in seeking treatment by bivariate analysis (n = 494)

Factors	Number (%)	Total delays		*P*
		Delays (%)	OR (95%CI)	
Gender (n = 494)				
Male	485 (98.2)	415 (85.6)	1.00	
Female	9 (1.8)	8 (88.9)	1.35 (0.17–10.96)	0.779
Age (years) (n = 494)				
< 30	113 (22.9)	96 (85.0)	1.00	
30–49	316 (64.0)	266 (84.2)	0.94 (0.52–1.71)	0.845
≥ 50	65(13.1)	61 (93.9)	2.70 (0.87–8.41)	0.086
Marital Status (n = 494)				
Married	394 (79.8)	347 (88.1)	1.00	
Single/divorce/separated	100 (20.2)	76 (76.0)	0.43 (0.25–0.74)	0.003
Occupation (n = 494)				
Professionals	89 (18.0)	73 (82.0)	1.00	
Workers	96 (19.4)	79 (82.3)	1.02 (0.48–2.16)	0.962
Farmers	170 (34.4)	157 (92.4)	2.65 (1.21–5.79)	0.015
Other	139 (28.2)	114 (82.0)	1.00 (0.50–2.00)	0.999
Residence (n = 494)				
Urban	185 (37.8)	261 (85.9)	1.00	
Rural	304 (62.2)	157 (84.9)	0.92 (0.55–1.55)	0.763
Region (n = 494)				
Eastern	67 (13.6)	55 (82.1)	1.00	
Central	309 (62.6)	259 (83.8)	1.13 (0.56–2.26)	0.730
Western	118 (23.9)	109 (92.4)	2.64 (1.05–6.65)	0.039
Malaria parasite type of patient infection (n = 494)				
* Plasmodium falciparum*	308 (62.4)	253 (82.1)	1.00	
* Plasmodium vivax/Plasmodium malariae*^*a*^	98 (19.8)	89 (90.8)	2.15 (1.02–4.53)	0.044
* Plasmodium ovale*	15 (3.0)	14 (93.3)	3.04 (0.39–23.63)	0.287
Mixed	73 (14.8)	67 (91.8)	2.43 (1.00–5.88)	0.049
Healthcare facilities for the first visit (n = 494)				
Village clinic/Township health center	148 (30.0)	142 (96.0)	1.00	
County-level health institutions	158 (32.0)	139 (88.0)	0.31 (0.12–0.80)	0.015
Prefectural health institutions	145 (29.3)	111 (76.6)	0.14 (0.06–0.34)	0.000
Provincial health institutions	43 (8.7)	31 (72.1)	0.11 (0.04–0.31)	0.000
Symptom (n = 494)				
Fever	491 (99.4)	422 (86.0)	12.23 (1.09–136.72)	0.042
Chills	209 (42.3)	183 (87.6)	1.32 (0.78–2.22)	0.295
Sweating	191 (38.7)	171 (89.5)	1.73 (1.00–3.01)	0.052
Splenomegaly	7 (1.4)	6 (85.7)	1.01 (0.12–8.49)	0.995
Anaemia	41 (8.3)	38 (92.7)	2.24 (0.67–7.45)	0.190
Periodic^b^	123 (24.9)	118 (95.9)	5.11 (2.01–12.99)	0.001
Insurance status (n = 494)				
Employee Basic Medical Insurance	70 (14.2)	59 (84.3)	1.00	
Resident Basic Medical Insurance	253 (51.2)	222 (87.8)	1.34 (0.63–2.81)	0.447
Self-payment	148 (30.0)	125 (84.5)	1.01 (0.46–2.22)	0.974
Other	23 (4.6)	17 (73.9)	0.53 (0.17–1.64)	0.269

^a^This grouping represents that individuals infected separately with *Plasmodium vivax* and *Plasmodium malariae* were merged into the same group

^b^Periodic interval episodes are one of the typical symptoms of malaria. The main clinical manifestations are periodic systemic chills, fever and hyperhidrosis during the onset of the disease. The intervals are normal and may last for several days

Significant factors influencing patient delays in seeking treatment identified by the bivariate analysis were: being single/divorce/separated, being a farmer, living in Central China, infection with *Plasmodium vivax*/*Plasmodium malariae*, mixed infections, first visiting a county-level health institution, first visiting a prefectural health institution, and first visiting a provincial health institution.

Significant factors influencing doctor delays in seeking treatment identified by the bivariate analysis were: age between 30 to 49 years old, living in Central China, living in Western China, mixed infections, first visiting a county-level health institution, first visiting a prefectural health institution, first visiting a provincial health institution, sweating, and having resident medical insurance (Table 2).

**Table 2:** Factors associated with patient delays and doctor delays in seeking treatment by bivariate analysis (n = 494)

Factors	Patient delays		p-value	Doctor delays		p-value
	Delays (%)	OR (95%CI)		Delays (%)	OR (95%CI)	
Gender (n = 494)						
Male	226 (46.6)	1.00		258 (53.2)	1.00	
Female	5 (55.6)	1.43 (0.38–5.40)	0.595	5 (55.6)	1.10 (0.29–4.15)	0.888
Age (years) (n = 494)						
< 30	50 (44.3)	1.00		69 (61.1)	1.00	
30–49	145 (45.9)	1.07 (0.69–1.65)	0.764	153 (48.4)	0.60 (0.39–0.93)	0.022
≥ 50	36 (55.4)	1.56 (0.85–2.89)	0.153	41 (63.1)	1.09 (0.58–2.05)	0.790
Marital status (n = 494)						
Married	196 (49.8)	1.00		216 (54.8)	1.00	
Single/divorce/separated	35 (35.0)	0.54 (0.35–0.86)	0.009	47 (47.0)	0.73 (0.47–1.14)	0.162
Occupation (n = 494)						
Professionals	37 (41.6)	1.00		47 (52.8)	1.00	
Workers	45 (46.9)	1.24 (0.69–2.22)	0.468	46 (47.9)	0.82 (0.46–1.47)	0.506
Farmers	96 (56.5)	1.82 (1.09–3.06)	0.023	98 (57.7)	1.22 (0.73–2.04)	0.457
Other	53 (38.1)	0.87 (0.50–1.49)	0.604	72 (51.8)	0.96 (0.56–1.64)	0.882
Residence (n = 494)						
Urban	143 (47.0)	1.00		173 (56.9)	1.00	
Rural	85 (46.0)	0.96 (0.66–1.38)	0.814	89 (48.1)	0.70 (0.49–1.01)	0.059
Region (n = 494)						
Eastern	40 (59.7)	1.00		24 (35.8)	1.00	
Central	113 (36.6)	0.39 (0.23–0.67)	0.001	173 (56.0)	2.28 (1.32–3.94)	0.003
Western	78 (66.1)	1.32 (0.71–2.45)	0.385	66 (55.9)	2.27 (1.23–4.22)	0.009
Malaria parasite type of patient infection (n = 494)						
* Plasmodium falciparum*	139 (45.1)	1.00		151 (49.0)	1.00	
* Plasmodium vivax/Plasmodium malariae*	62 (63.3)	2.09 (1.31–3.34)	0.002	52 (53.1)	1.18 (0.75–1.85)	0.487
* Plasmodium ovale*	8 (53.3)	1.39 (0.49–3.93)	0.535	7 (46.7)	0.91 (0.32–2.57)	0.858
Mixed	22 (30.1)	0.52 (0.30–0.91)	0.021	53 (72.6)	2.76 (1.57–4.83)	0.000
Healthcare facilities for the first visit (n = 494)						
Village clinic/Township health center	–	1.00		127 (85.8)	1.00	
County-level health institutions	–	2.42 (1.50–3.90)	0.000	90 (57.0)	0.22 (0.13–0.38)	0.000
Prefectural health institutions	–	4.27 (2.61–6.99)	0.000	37 (25.5)	0.06 (0.03–0.10)	0.000
Provincial health institutions	–	3.30 (1.64–6.65)	0.001	9 (20.9)	0.04 (0.02–0.10)	0.000
Symptom (n = 494)						
Fever	230 (46.8)	1.76 (0.16–19.56)	0.644	262 (53.4)	2.29 (0.21–25.40)	0.500
Chills	93 (44.5)	0.85 (0.60–1.22)	0.388	108 (51.7)	0.90 (0.63–1.28)	0.551
Sweating	91 (47.6)	1.06 (0.74–1.52)	0.755	116 (60.7)	1.64 (1.14–2.37)	0.008
Splenomegaly	1 (14.3)	0.19 (0.02–1.56)	0.121	6 (85.7)	5.37 (0.64–44.93)	0.121
Anaemia	19 (46.3)	0.98 (0.52–1.86)	0.955	26 (63.4)	5.37 (0.64–44.93)	0.121
Periodic	58 (47.2)	1.02 (0.68–1.54)	0.920	78 (63.4)	5.37 (0.64–44.93)	0.121
Insurance status (n = 494)						
Employee basic medical insurance	36 (51.4)	1.00		30 (42.9)	1.00	
Resident basic medical insurance	117 (46.3)	0.81 (0.48–1.38)	0.442	153 (60.5)	2.04 (1.19–3.49)	0.009
Self-payment	67 (45.3)	0.78 (0.44–1.38)	0.396	71 (48.0)	1.23 (0.69–2.18)	0.480
Other	11 (47.8)	0.87 (0.34–2.22)	0.764	9 (39.1)	0.86 (0.33–2.24)	0.753

In the multiple logistic regression analysis, the factors significantly associated with total delays in seeking treatment were: being single/divorce/separated (OR = 0.44, 95% CI 0.20–0.96), being a farmer (OR = 3.88, 95% CI 1.38–10.95), first visiting a county-level health institution (OR = 0.25, 95%CI 0.09–0.69), first visiting a prefectural health institution (OR = 0.14, 95%CI 0.05–0.40), first visiting a provincial health institution (OR = 0.13, 95%CI 0.04–0.42), and having periodic symptoms (OR = 5.70, 95% CI 2.06–15.81) (Table 3).

**Table 3:** Factors associated with three types of delays in seeking treatment by multiple logistic regression (n = 494)

Factors	Total delays	p-value	Patient delays	p-value	Doctor delays	p-value
	OR (95%CI)		OR (95%CI)		OR (95%CI)	
Gender (n = 494)						
Male	1.00		1.00		1.00	
Female	0.72 (0.07–7.15)	0.782	1.15 (0.24–5.50)	0.858	2.38 (0.48–11.77)	0.288
Age (years) (n = 494)						
< 30	1.00		1.00		1.00	
30–49	0.70 (0.31–1.58)	0.390	0.94 (0.54–1.63)	0.832	0.43 (0.23–0.81)	0.009
≥ 50	1.74 (0.47–6.47)	0.408	1.25 (0.60–2.60)	0.551	0.77 (0.33–1.80)	0.546
Marital status (n = 494)						
Married	1.00		1.00		1.00	
Single/divorce/separated	0.44 (0.20–0.96)	0.038	0.69 (0.38–1.24)	0.210	0.48 (0.24–0.95)	0.035
Occupation (n = 494)						
Professionals	1.00		1.00		1.00	
Workers	1.44 (0.60–3.48)	0.420	1.22 (0.63–2.34)	0.558	1.17 (0.55–2.52)	0.684
Farmers	3.88 (1.38–10.95)	0.010	1.76 (0.88–3.49)	0.108	1.05 (0.48–2.34)	0.898
Other	1.82 (0.76–4.38)	0.181	1.01 (0.53–1.90)	0.989	1.43 (0.68–3.00)	0.340
Residence (n = 494)						
Urban	1.00		1.00		1.00	
Rural	2.06 (1.00–4.26)	0.051	1.12 (0.69–1.83)	0.651	1.12 (0.64–1.98)	0.694
Region (n = 494)						
Eastern	1.00		1.00		1.00	
Central	1.10 (0.49–2.49)	0.819	0.43 (0.24–0.78)	0.006	1.59 (0.81–3.13)	0.179
Western	2.10 (0.68–6.44)	0.195	1.30 (0.62–2.75)	0.487	1.39 (0.60–3.20)	0.444
Malaria parasite type of patient infection (n = 494)						
* Plasmodium falciparum*	1.00		1.00		1.00	
* Plasmodium vivax/Plasmodium malariae*	1.09 (0.43–2.76)	0.861	1.29 (0.71–2.35)	0.404	0.82 (0.42–1.58)	0.546
* Plasmodium ovale*	2.00 (0.22–17.79)	0.536	1.06 (0.34–3.28)	0.921	0.54 (0.15–1.92)	0.338
Mixed	2.02 (0.76–5.38)	0.161	0.61 (0.34–1.10)	0.101	2.04 (1.02–4.08)	0.043
Healthcare facilities for the first visit (n = 494)						
Village clinic / Township health center	1.00		–	–	1.00	
County-level health institutions	0.25 (0.09–0.69)	0.008	–	–	0.25 (0.14–0.45)	0.000
Prefectural health institutions	0.14 (0.05–0.40)	0.000	–	–	0.06 (0.03–0.12)	0.000
Provincial health institutions	0.13 (0.04–0.42)	0.001	–	–	0.05 (0.02–0.12)	0.000
Symptom (n = 494)						
Fever	8.99 (0.53–151.54)	0.128	1.28 (0.11–15.63)	0.845	1.11 (0.08–15.46)	0.937
Chills	0.95 (0.51–1.76)	0.864	0.70 (0.46–1.06)	0.094	0.75 (0.47–1.20)	0.231
Sweating	0.76 (0.39–1.48)	0.420	0.72 (0.47–1.12)	0.146	0.99 (0.60–1.63)	0.958
Splenomegaly	0.32 (0.03–3.74)	0.364	0.19 (0.02–1.72)	0.137	3.50 (0.28–44.55)	0.335
Anaemia	2.21 (0.57–8.66)	0.254	1.47 (0.71–3.01)	0.297	1.26 (0.55–2.93)	0.585
Periodic	5.70 (2.06–15.81)	0.001	1.36 (0.85–2.18)	0.195	1.71 (1.00–2.92)	0.049
Insurance status (n = 494)						
Employee basic medical insurance	1.00		1.00		1.00	
Resident basic medical insurance	1.02 (0.38–2.71)	0.969	0.79 (0.41–1.55)	0.499	1.62 (0.75–3.49)	0.222
Self-payment	0.99 (0.40–2.41)	0.978	0.79 (0.42–1.48)	0.456	1.52 (0.74–3.14)	0.256
Other	0.64 (0.18–2.28)	0.490	0.97 (0.35–2.67)	0.950	1.10 (0.32–3.80)	0.879

The factor significantly associated with patient delays in seeking treatment was living in Central China (OR = 0.43, 95% CI 0.24–0.78). The factors significantly associated with doctor delays in seeking treatment were: age between 30 to 49 years old (OR = 0.43, 95% CI 0.23–0.81), being single/divorce/separated (OR = 0.48, 95% CI 0.24–0.95), mixed infections (OR = 2.04, 95%CI 1.02–4.08), first visiting a county-level health institution (OR = 0.25, 95%CI 0.14–0.45), first visiting a prefectural health institution (OR = 0.06, 95%CI 0.03–0.12), first visiting a provincial health institution (OR = 0.05, 95%CI 0.02–0.12), and having periodic symptoms (OR = 1.71, 95% CI 1.00–2.92).

### Type and duration of delays in healthcare-seeking

Table 4 presented an overview of the characteristics of malaria cases from the time of symptoms onset to the conclusive diagnosis. Out of the 494 malaria patients, only 71 patients (14.4%) were conclusively diagnosed and treated within 1 day after the onset of symptoms. indicating that 85.6% of patients experienced delays. The majority (261/494, 52.8%) had total delays of more than 3 days. The median time from the onset of symptoms to the initial healthcare consultation was 1 day. Approximately half (263/494, 53.2%) started to seek healthcare consultation within 1 day after symptom onset, while nearly one-fifth (89/494, 18.0%) initiated medical visits more than 3 days later. The median time from the initial healthcare consultation to the conclusive diagnosis was 2 day. After the initial visit, 46.8% of patients received a conclusive diagnosis within 1 day, while 33.2% experienced doctor delays of more than 3 days. Overall, delays solely attributed to patients accounted for 28.3%, while delays solely attributed to doctors accounted for 34.8%. There were 91 cases (18.4%) with both types of delays, while the remaining 91 cases experienced no delay at all.

**Table 4:** Characteristics of healthcare-seeking behavior from the time of symptoms onset to the conclusive diagnosis

Characteristics of healthcare-seeking behavior (n = 494)	Number (%)
The total delays from symptoms onset to conclusive diagnosis	
Within 24 h	71 (14.4)
From 24 to 48 h	74 (15.0)
From 48 to 72 h	88 (17.8)
Longer than 3 days	261 (52.8)
The patient delays from symptoms onset to initial healthcare consultation	
Within 24 h	263 (53.2)
From 24 to 48 h	70 (14.2)
From 48 to 72 h	72 (14.6)
Longer than 3 days	89 (18.0)
The doctor delays from initial healthcare consultation to conclusive diagnosis	
Within 24 h	231 (46.8)
From 24 to 48 h	59 (11.9)
From 48 to 72 h	40 (8.1)
Longer than 3 days	164 (33.2)
Types of delays	
None delay	91 (18.4)
Patient delays only	140 (28.3)
Doctor delays only	172 (34.8)
Both patient and doctor delays	91 (18.4)

As shown in Table 5, both patient delays and doctor delays increased the financial burden on malaria patients (≥ 1018 USD) and made complications more likely. In addition to incurring these consequences, doctor delays resulted in longer hospital stays (≥ 6 days).

**Table 5:** Characteristics of patient delays and doctor delays on outcome variables

Outcome variables	Patient delays		p-value	Doctor delays		p-value
	Yes (%)	No (%)		Yes (%)	No (%)	
Economic burden (n = 494)						
< 1018 USD	134 (55.1)	109 (44.9)	0.000	106 (43.6)	137 (56.4)	0.000
≥ 1018 USD	97 (38.7)	154 (61.3)		157 (62.6)	94 (37.4)	
Length of stay (n = 494)						
< 6 days	73 (48.0)	79 (52.0)	0.707	70 (46.1)	82 (54.0)	0.033
≥ 6 days	158(46.2)	184 (53.8)		193 (56.4)	149 (43.6)	
Complications developed (n = 494)						
Yes	32 (34.4)	61 (65.6)	0.008	60 (64.5)	33 (35.5)	0.016
No	199 (49.6)	202 (50.4)		203 (50.6)	198 (49.4)	

**Fig. 1:**
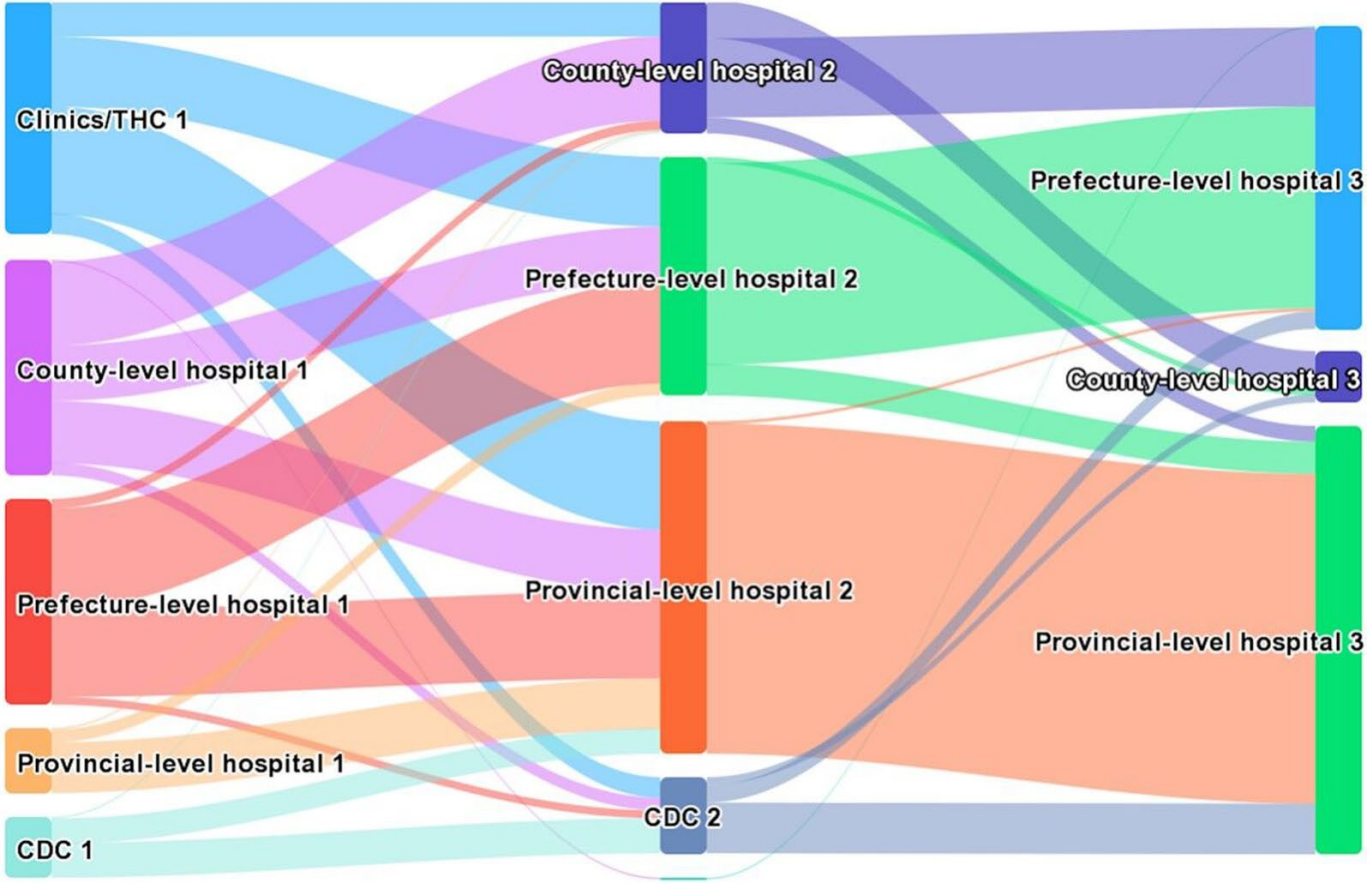
Trajectories of malaria patients seeking health care

In the Sankey diagram, the three types of nodes represented, from left to right, health institutions for the first visit, health institutions that malaria patients were confirmed, and the conclusive hospitals that report malaria cases, as shown in Fig 1. The width of the branch indicated the magnitude of the flow, so the proportion of patients in different segments could be compared visually. The referral flow of malaria cases generally conformed to the rule from primary health institutions to higher-level health institutions. In the sample, 284 (57.5%) of the malaria patients initially sought medical treatment at a village clinic/township health center or a county hospital, and then they were transferred to the same or higher level of health institutions for further diagnosis and treatment. In the end, only 6.7% of the patients stayed in county hospitals. The CDC mainly played a diagnostic role in malaria treatment, from which more than two-thirds of cases went to provincial hospitals with better medical conditions.During the referral process, the number of malaria cases admitted to prefectural hospitals continued to increase, with the proportion climbing from 26.3% to 38.7%. The number of cases admitted to provincial hospitals increased sharply, from only 41 cases (8.3%) at the first visit to 270 cases (54.6%) eventually.

## Discussion

Although the NMEAP was launched as early as 2010, calling for malaria patients to receive prompt and accurate treatment, it is still far from achieving this goal. In this study, 85.6% of malaria patients experienced total delays of more than 1 day after symptoms onset. This study explored the health-seeking behaviours and influencing factors of treatment delays among malaria patients in 6 typical provinces of China. It found that the level of health institutions where malaria patients initially visited was crucial for the early diagnosis and timely treatment. Furthermore, most malaria patients were correctly diagnosed and treated during multiple referrals to higher-level hospitals for further medical services.

From the initial visit to the conclusive diagnosis, 46.8% of patients spent time within 1 day, indicating that more than half (53.2%) of malaria cases encountered doctor delays. The prevalence of such delays deserves great attention from the society. The results showed that patients aged 30 to 49 were less likely to experience doctor delays. In this age group, many patients may be better equipped to describe malaria symptoms and cooperate with treatment, making it easier for doctors to make a correct diagnosis [24]. Single/divorced/separated patients were less prone to doctor delays. The reason may be that these individuals are generally more independent and communicate more directly with the medical team [25]. However, more detailed explanation for this association is still lacking. The first visit to a county-, prefectural-, and provincial-level medical institutions were also negatively associated with doctor delays. These institutions have more sufficient medical resources and more advanced technical equipment [26]. Patients treated here are easier to obtain health knowledge about malaria, potentially reducing the risk of doctor delays. Moreover, mixed infections with malaria can lead to more complex symptoms, and periodic symptoms appear and disappear at different times, so it is difficult to diagnose the disease correctly [27]. Doctors may also misinterpret these symptoms as other common illnesses, such as the flu or the common cold, increasing the likelihood of doctor delays. The results also showed that living in central China were less likely to experience patient delays. Previous studies have demonstrated that emphasis on malaria awareness and education in this region [28]. This may contribute to patients take the risk of malaria seriously.

Therefore this study suggests that government decision-makers need to scale up malaria health education interventions. The target is not only to improve the awareness among health care personnel in responding to imported malaria [29, 30], but also to increase the knowledge of at-risk populations traveling to malaria-prone areas such as sub-Saharan Africa and Southeast Asia [31]. Internet-based health education channels have been extensively utilized in recent years. Digital media have been proven to be an effective, feasible and widely accepted strategy for improving health, which can be considered as a means of communication for health education in malaria [32, 33].

With regard to malaria treatment outcomes, delays can lead to disease deterioration, which also resulted in longer treatment time and greater economic burden. Complications in malaria patients necessitate extended hospitalization and supervision until a stable state is achieved. Additionally, other evidences showed that, based on the comparison between average hospitalization costs and average income, the rude estimation of malaria treatment costs might be beyond the reach of rural households [34–36]. This highlights the critical role of timely medical treatment in the effective management of malaria.

The flow of malaria patients clearly revealed that they were inclined to choose primary health institutions closer to their homes for the first visit. Combined with many previous studies, the reasons can be explained as that most of the imported malaria patients are overseas workers, who are less aware of malaria prevention and treatment [28, 37–39]. Moreover, the medical treatment decisions are constrained by economic conditions, so primary health institutions with low prices are the preferred choice of patients. However, thanks to the insufficient stock of antimalarial drugs in primary care settings and the low level of knowledge of doctors, it is easy to cause treatment delays and misdiagnosis [40]. This is particularly concerning for malaria patients with severe illness or high-risk factors, as it significantly elevates the likelihood of disease progression. Hence, an important prerequisite for appropriate health care-seeking behaviour among malaria patients is that there should be no significant difference in the effects of medical services provided by all levels of health institutions. Improving the malaria diagnosis and treatment capabilities of primary health institutions plays a vital role in directing the flow of malaria patients to the rational place [21]. This study suggests that government decision-makers need not only to enhance medical techniques of primary health institutions but also to ensure seamless access to health services at higher administrative levels. To address this, it is imperative to establish a rapid referral mechanism between primary health institutions and higher-level hospitals designated for malaria treatment. This ensures that suspected malaria cases have access to effective and high-precision malaria tests and therapeutic drugs within a short period of time. Such a mechanism is crucial for minimizing delays in diagnosis and treatment, especially for severe malaria cases.

## Limitations of the study

There are certain limitations in the study. Firstly, the exclusion of cases from hospitals unwilling to provide data and cases with incomplete information might have impacted the sample size. However, a larger sample size would have strengthened the analysis results and increased their persuasiveness. Secondly, the calculation of delays in seeking treatment was not precise to the hour. Patient medical records only provided the dates of symptoms onset and the dates of visit to a health institution or CDC, without specific time periods. Consequently, this may have resulted in an overestimation of delays in the statistics than the actual situation. Thirdly, due to policy restrictions on the availability of medical information resources in China, this study was conducted retrospectively between 2014 and 2016, which may not reflect the current situation. Nonetheless, it is crucial to note that this study was conducted during the final phase of malaria elimination. The factors influencing delays in seeking treatment may offer valuable insights for formulating intervention policies in other countries facing similar challenges to China.

## Conclusions

There was a substantial delay in access to health care for malaria patients in the final phase of malaria elimination. Region, marital status, periodic symptoms and the level of health institutions were factors contributing to delays in treatment-seeking among malaria patients. This study recommends strengthening health awareness among people at risk of imported malaria and improving the diagnostic capacity of primary health institutions to reduce delays from both doctors and patients.

### Supplementary Information

**Additional file 1: Figure S1.** Geographic distribution of the six selected provinces in China.

## Data Availability

The dataset analyzed during the current study are available from the corresponding authors on reasonable request.

## Abbreviations

WHO: World Health Organization
NMEAP: National Malaria Elimination Action Programme
RDTs: Rapid diagnostic tests
nPCR: Nested polymerase chain reaction
CDCs: Centers for Disease Control and Prevention
