# Supplementary figures and images for: Factors affecting delays in seeking treatment among malaria patients during the pre-certification phase in China
Source: Malar J. 2024 Mar 11;23:73. doi: 10.1186/s12936-024-04892-4 (PMC10929211; doi:10.1186/s12936-024-04892-4)

| 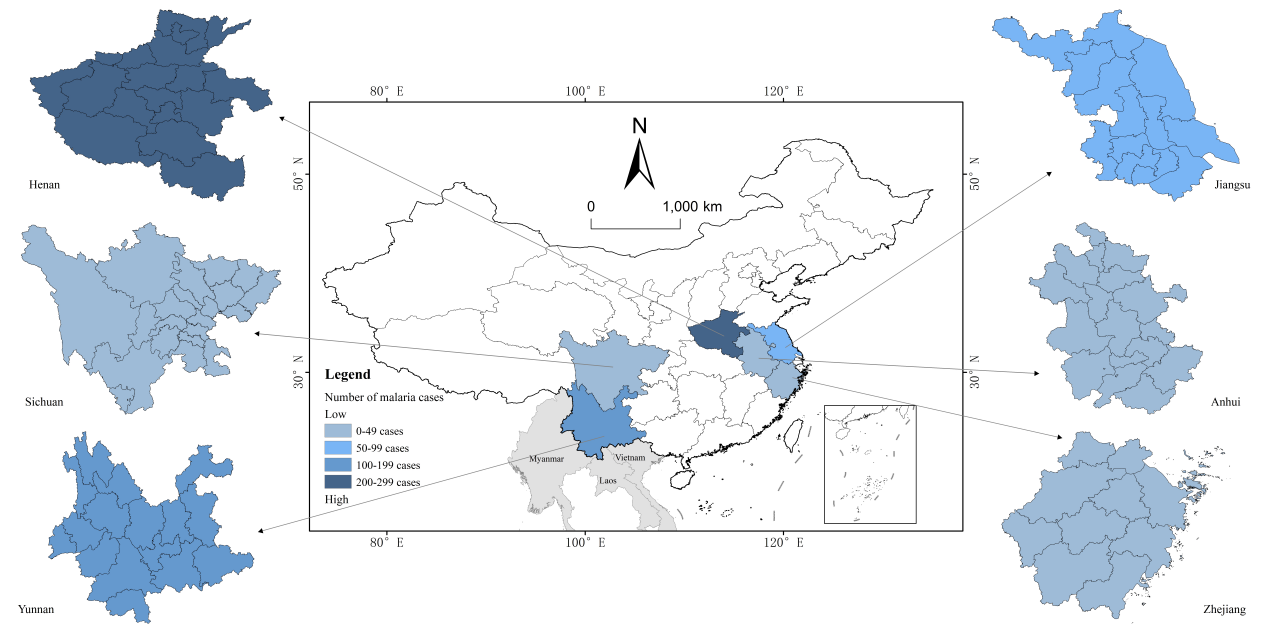 |
| --- |
| Figure 1 Geographic distribution of the six selected provinces in China |

Supplement: Supplementary file 1 — Additional file 1: Figure S1. Geographic distribution of the six selected provinces in China. [file 12936_2024_4892_MOESM1_ESM.docx]
